# Microvascular Complications and Their Associated Risk Factors in Newly Diagnosed Type 2 Diabetes Mellitus Patients

**DOI:** 10.1155/2014/201423

**Published:** 2014-11-30

**Authors:** Dipika Bansal, Kapil Gudala, Hari Prasad Esam, Ramya Nayakallu, Raja Vikram Vyamusani, Anil Bhansali

**Affiliations:** ^1^Department of Pharmacy Practice, National Institute of Pharmaceutical Education and Research, Mohali 160062, India; ^2^Department of Endocrinology, Postgraduate Institute of Medical Education and Research, Chandigarh 160062, India

## Abstract

The study was aimed at assessing the prevalence of microvascular complications and associated risk factors in newly diagnosed type 2 diabetes mellitus patients. A cross-sectional study was conducted in a public tertiary care hospital. All the recruited patients underwent extensive examination for the presence of microvascular complications like neuropathy, retinopathy, and nephropathy. Prevalence of any complication was 18.04%. Prevalence of neuropathy, retinopathy, and nephropathy was found to be 8.2%, 9.5%, and 2.8%, respectively. Triglycerides (OR, 1.01; *P* = 0.011) and old age (OR, 1.06; *P* ≤ 0.01) were significantly associated with any complication. Triglycerides were significantly associated with neuropathy (OR, 1.01; *P* = 0.05) and retinopathy (OR, 1.01; *P* = 0.02). Being male posed high risk for nephropathy (OR, 0.06; *P* = 0.01). These results are suggesting need of regular screening for microvascular complications.

## 1. Introduction

Type 2 diabetes mellitus (T2DM) has become a global burden; about 382 million people are diagnosed with diabetes mellitus (DM) with an annual prevalence of 8.2% [1]. India is the second largest country in terms of DM burden with 65.1 million diagnosed cases [1].

T2DM is characterized by an asymptomatic phase between the actual onset of diabetic hyperglycemia and clinical diagnosis. The onset of T2DM is usually subtle and many years may elapse before diagnosis. This asymptomatic phase is estimated to last at least 4–7 years and consequently 30–50% patients may remain undiagnosed [2]. This becomes more imperative because of limited health resources and inadequate budget allocation to health. T2DM may actually be detected at the time of diagnosis of its complications. Microvascular complications from T2DM are common and evidence shows that early detection and identification of risk factors for retinopathy, nephropathy, and neuropathy may delay or prevent the progression towards blindness, end-stage renal disease, and diabetic foot ulcers, respectively [3]. Long-standing untreated hyperglycemia is responsible for the relatively high prevalence of microvascular complications in newly diagnosed diabetes mellitus (NDDM) patients [2]. Presence of microvascular complications at the time of T2DM diagnosis are showing increasing trend in India.

It is apparent that evidence on prevalence of T2DM related complications is essential for the adjustment of policies and practices in diabetic care management. Screening for microvascular complications in NDDM patients will have important implications for understanding the need of vigorous screening, effective prevention, and management of T2DM as well as reduced healthcare expenditure.

Present study is aimed at assessing the prevalence and risk factors of microvascular complications in NDDM patients of a public tertiary care hospital in India.

## 2. Materials and Methods

### 2.1. Study Design and Setting

A prospective, cross-sectional, single centre, interview based study was conducted between July 2011 and June 2013 in the endocrinology outpatient department of a public tertiary care hospital located in Chandigarh, India. The study was initiated after obtaining approval from the Institute Ethical Committee (IEC, PGIMER, Chandigarh, India).

### 2.2. Subject Recruitment

Consecutive outpatient cards were screened to recruit eligible subjects. Subjects of either gender newly diagnosed with T2DM (≤6 months of duration) [4] as per American Diabetes Association (ADA) guidelines (random plasma glucose > 200 mg/dL or fasting blood sugar > 126 mg/dL or HbA1c ≥ 6.5) [5] and willing to give prior informed consent were included in the study. All NDDM subjects were required to undergo an extensive medical examination for the assessment of microvascular complications.

### 2.3. Variables and Data Sources

Anthropometric measurements including weight, height (using stadiometer), body mass index (BMI; kg/m^2^), and waist circumference (using inelastic and flexible tape at the midpoint between the lower margin of the least palpable rib and top of the iliac crest nearest to 0.1 cm) were carried out at the time of recruitment. Information about socioeconomic and lifestyle characteristics (smoking and alcohol consumption) was obtained through patient interview at the time of recruitment. Modified Kuppuswamy's scale [6] which includes the educational qualification, occupation, and monthly family income of the subject was used to assess the socioeconomic status. Clinical systolic blood pressure (SBP) and diastolic blood pressure (DBP) levels, serum lipids, blood glucose and glycated hemoglobin (HbA1c), and hepatic and renal function levels were extracted from available clinical records (in the previous 3 months).

Blood pressure was measured in the sitting position in right arm to the nearest 2 mmHg with a mercury sphygmomanometer (Diamond Deluxe BP apparatus, BP Instruments, Pune, India), and the participants were considered to be hypertensive if they were taking antihypertensive medication (as documented in clinic records) or SBP ≥ 140 mmHg or DBP ≥ 90 mmHg. HbA1c was measured using the Variant machine (Bio-Rad Laboratories, Hercules, CA, USA). Serum cholesterol (cholesterol esterase oxidase-peroxidase-amidopyrine method), serum triglycerides (glycerol phosphate oxidase-peroxidase-amidopyrine method), and high density lipoprotein cholesterol (direct method polyethylene-glycol-pretreated enzymes) were measured using the Beckman Coulter AU 2700/480 Autoanalyser (Beckman AU (Olympus), Ireland).

### 2.4. Microvascular Complications

Assessment of neuropathy was done using 10 gm Von Frey monofilament (VMF), pinprick sensations, ankle reflexes, and vibration perception threshold (VPT). 10 gm VMF was located perpendicular to the skin and pressure was applied until the filament just bends with a contact time of 2 seconds. Inability to comprehend the sensation at any site was considered abnormal. In addition, presence or absence of ankle reflex was checked using percussion hammer. Then quantification of neuropathy was done by biothesiometer (Dhansai Laboratories, Mumbai, India); it was measured at five different locations of feet (distal plantar surface and metatarsals) of both legs. The voltage was slowly increased at the rate of 1 millivolt per second (mV/sec) until subject indicated that he or she has felt the first vibration sense. The mean value of five measurements of both legs was calculated and considered for analysis. Neuropathy was considered to be mild if the VPT reading was found between 20 and 24 mV, moderate (25–39 mV), and severe (>39 mV) [7]. Initially, each diabetic patient was confirmed by the physician to have DPN if diagnosed with one or more abnormal finding of 10 gram VMF, pinprick sensations, and ankle reflexes. Thereafter, the patient underwent VPT testing to categorize them according to the severity level of DPN.

The diagnosis of retinopathy was confirmed from clinical records (if already documented) or sent for extensive ophthalmologic examination that included fundoscopy or retinal photography and measurement of visual acuity, performed by an ophthalmologist. They were classified into proliferative diabetic retinopathy (PDR) or nonproliferative diabetic retinopathy (NPDR) accordingly [8].

The diagnosis of nephropathy was confirmed by estimating 24 hours urine protein excretion of more than 500 mg/day [9].

### 2.5. Statistical Analysis

Data was presented as mean and standard deviation (SD) or median with interquartile range and numbers with percentages. Data was analyzed using either two-sample independent student *t*-test or Mann-Whitney test and *χ* ^2^ tests. The variables like age, gender, BMI, smoking status, alcoholic status, and biochemical parameters were considered as risk factors. Multivariate logistic regression was performed to estimate odds ratios (OR) for assessing the risk factors associated with presence of microvascular complications with 95% confidence interval (CI). Two-tailed *P* value of less than 0.05 was considered as significant. Prescribing pattern of different antidiabetic drugs was also reported in the form of percentages. All the analyses were carried out using SPSS version 14 (SPSS Inc., Chicago, IL, USA).

## 3. Results

### 3.1. Patient Characteristics

A total of 449 NDDM subjects were included in the study. Among them were 206 (46%) males and 243 (54%) females with mean age of 50.4 ± 10.3 years and median duration of diabetes of 2.4 (0.96–4.8) months. A total of 81 (18.04%) patients were found to have at least one microvascular complication and none had any history of macrovascular complication.  Table 1 shows clinical and biochemical characteristics based on the presence of microvascular complications. Subjects with microvascular complications were older (*P* < 0.001) and were having significantly (*P* < 0.001) higher HbA1c values. Higher triglyceride (*P* = 0.005) levels were also observed in subjects with microvascular complications.

**Figure 1:**
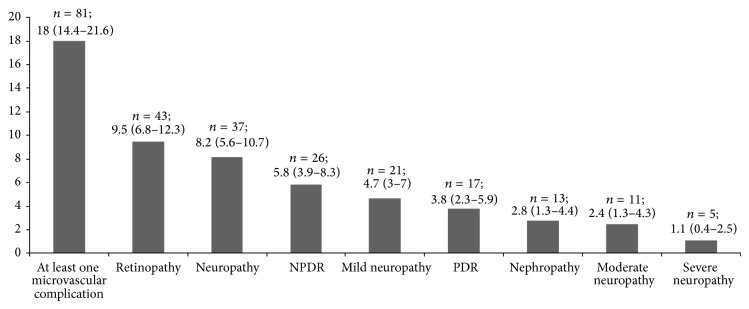
Prevalence of microvascular complications in newly diagnosed diabetes mellitus patients (*n* = 449).

In Figure 1 prevalence of microvascular complications is presented. The prevalence of any microvascular complication was 18.04% (95% CI, 14.4–21.6). Neuropathy was found to be 8.2% (95% CI, 5.6–10.7), retinopathy was 9.5% (95% CI, 6.8–12.3), and nephropathy was 2.8% (95% CI, 1.3–4.4). Vibration perception threshold revealed that 4.7% (95% CI, 3–7) were having mild neuropathy, 2.4% (95% CI, 1.3–4.3) were having moderate neuropathy, and 1.1 (95% CI, 0.4–2.5) were having severe neuropathy. Among patients of retinopathy, 5.8% (95% CI, 3.9–8.3) were having NPDR and 3.8% (95% CI, 2.3–5.9) were having PDR.

Table 2 summarises the risk factors for presence of microvascular complications. The risk factors for having at least one microvascular complication were found to be age (OR, 1.06; 95% CI, 1.03–1.08, *P* < 0.01), HbA1c (OR, 1.24; 95% CI 1.12–1.37, *P* = 0.047), and triglycerides (OR, 1.01; 95% CI, 1.00–1.02, *P* = 0.011).

Age (OR, 1.08; CI, 1.04–1.12; *P* < 0.01) and triglycerides (OR, 1.01; CI, 1.00–1.05; *P* = 0.05) were found to be risk factors for neuropathy. While retinopathy was classified into NPDR and PDR, NPDR was found to be associated with HbA1c (OR, 1.33; CI, 1.16–1.53; *P* = 0.045) and triglycerides (OR, 1.01; CI, 1.00–1.02; *P* = 0.02). PDR was significantly associated with only HbA1c (OR, 1.88; CI 1.13–2.34; *P* = 0.03). Female gender (OR, 0.06; CI, 0.01–0.51; *P* = 0.01) is negatively associated with nephropathy whereas higher levels of triglycerides (OR, 1.01; CI, 1.00–1.02; *P* = 0.032) and serum creatinine (OR, 1.55; CI, 1.26–2.59; *P* = 0.047) were positively associated with nephropathy (Table 2). Nephropathy was found to be dependent on retinopathy (OR, 9.2; CI, 2.9–28.9; *P* ≤ 0.001) (data not shown in table).

Prescribing pattern of antidiabetic medication and medication for other comorbidities is presented in Table 3. In NDDM patients biguanides (74%) were the most commonly prescribed antidiabetic drugs both as monotherapy 39% (175) and as polytherapy 35% (158) followed by sulfonylureas 39% (176), insulin 37% (167), and thiazolidinediones 6% (29).

Overall, 80% (359) of patients were prescribed one or more oral hypoglycemic agents (OHA) while 37% (167) were prescribed insulin alone or in combination with OHA. Among them, 11% (47) of patients received only insulin while 27% (120) received insulin in combination with OHA. Further, 44% (196) were prescribed OHA monotherapy, 33% (147) were prescribed dual therapy, and 4% (16) were prescribed poly-OHA therapy. Biguanides were given in combination with sulfonylureas in 133 (30%) patients, followed by biguanides and thiazolidinediones in 8 (2%) and sulfonylureas and thiazolidinediones in 5 (1%) NDDM patients. 176 (39%) patients were prescribed with antihypertensives and 29 (6%) were prescribed with antiplatelet and 47 (10%) of subjects with lipid lowering drugs.

## 4. Discussion

T2DM is a complex disease, associated with long preclinical asymptomatic phase during which patients get exposed to long-standing persistent hyperglycemia before they are clinically diagnosed. This time lag between the onset of T2DM and clinical diagnosis results in the development of chronic micro- and macrovascular complications [2, 10]. In this study, we assessed the prevalence of microvascular complications in 449 NDDM patients and found 18.04% presented with at least one microvascular complication. Harris et al. showed that the onset of newly diagnosed T2DM probably occurs even earlier than 4–7 yr before clinical diagnosis [2, 10]. Based on our study results this proposition might be higher in developing country like India.

Similar cross-sectional studies have been done in India reporting prevalence rates ranging from 13 to 30% [11–14]. Raman et al. studied 248 newly diagnosed T2DM patients and reported a prevalence of 30.2% in south Indian population [11]. Studies by Patel et al. and Dutta et al. reported a prevalence rate of about 30% [12, 13]. The present study has reported low prevalence rates when compared to abovementioned studies. However, our study results are similar to a recently published multicentre observational study from India conducted by Sosale et al. who reported 13.15% of neuropathy, 6.1% of retinopathy, and 1.06% of nephropathy [14].

This variability in the prevalence may be due to difference in age at T2DM diagnosis, sample size, existing diagnostic facilities, and/or variable diagnostic criteria [15] followed by the studies.

Study conducted by Raman et al. used vibration perception threshold as the sole diagnostic measure for the assessment of neuropathy which might overestimate the prevalence of neuropathy [11]. Small sample size of studies by Azura et al. (*N* = 240), Raman et al. (*N* = 248), Dutta et al. (*N* = 100), and Patel et al. (*N* = 50) renders high chance of bias [11–13, 15]. This study also revealed that about 43% of subjects suffer from moderate to severe neuropathy which need immediate attention as they are at high risk of foot infection and amputation. Higher prevalence of retinopathy (9.5%) was also observed in the present study, which is higher than the prevalence observed in other studies conducted in India, Sosale et al. [14] and Raman et al. [11], and European study by de Fine Olivarius et al. (5%) [16]. Retinopathy is a common complication of diabetes and is usually the first observable vascular condition specific to diabetes. Untreated hyperglycemia may be one of the reasons for high prevalence of retinopathy in newly diagnosed T2DM subjects [10]. Relatively low prevalence of nephropathy was found (2.8%), as observed by Khazai et al. (3%) [17] and contrary to the results of Raman et al. [11], who has shown higher prevalence (10.5%). It is difficult to identify the reasons for such variation in prevalence rates among various populations but ethnic susceptibility, age, method of detecting diabetic complications, healthcare facilities, and other risk factors could have contributed to the differences.

Higher mean levels of HbA1c were observed in the present study, as the data was taken at the point of diagnosis of T2DM and the patients were newly initiated with the therapy that needs optimum time to show its effect on HbA1c levels. Present study findings suggest that advancing age, higher HbA1c, and triglyceride levels were risk factors for presence microvascular complications.

Various study findings also reported that microvascular complications increase with advancing age [18–20]. Similar cross-sectional studies by Kumar et al. found the relationship between triglycerides and presence of microvascular complications [21]. Aging and triglyceride levels were identified as independent risk factors for neuropathy. A randomized control clinical trial by Wiggin et al. [22] and few cross-sectional studies also reported similar relationship between triglycerides and neuropathy as shown in our study results [23, 24]. The relationship between triglycerides and neuropathy was first correlated in 1971; after that very few studies have shown the positive relation between triglycerides and neuropathy. The exact underlying mechanism behind the progression of neuropathy in relation to elevated triglycerides is yet to be clarified, but it may be due to dysregulation of lipid metabolism in sensory and motor neurons [22].

According to our findings, retinopathy and nephropathy were strongly correlated with each other similar to previous studies [25–28]. This finding may be helpful in adopting clinical significance of retinopathy as a strong predictor of nephropathy. Triglycerides were shown as risk factors in the present study, which strengthen the evidence of existing studies [29, 30].

In this study, we found that male gender and elevated triglyceride levels were the risk factors for the development of any one of the microvascular complications. A study conducted in T2DM subjects by Alrawahi et al. in Oman has also shown positive relationship with male gender [29]. Renoprotective action of estrogens may be responsible for lower incidence rate of nephropathy in females. However, existing literature also showed that renoprotective property of estrogens decreases due to imbalance of sex hormone regulation in T2DM females [31]. On the other hand previous studies have shown higher levels of triglycerides in diabetic nephropathy subjects [32–34]. Thus, it is also postulated that lipid induced renal injury may occur by stimulating TGF-*β* (transforming growth factor-beta), thereby inducing the production of reactive oxygen species causing damage to the glomeruli and glomerular glycocalyx [35].

*Limitations*. It is a tertiary care hospital based study so the prevalence of microvascular complications may be overestimated and may not match with community based studies. The inherent advantage is that the diagnosis performed in the study was by experienced endocrinologist, neurologist, and ophthalmologist so the chance of diagnosis error is minimal.

## 5. Conclusions and Future Implications

The present study reconfirms that a substantial proportion of patients which had clinically significant morbidity is present at diagnosis and for years before diagnosis of diabetes and its complications. Our study showed higher prevalence of retinopathy, followed by neuropathy and nephropathy; apart from glycemic control there is a need of tight lipid management in T2DM patients as triglycerides were shown as significant risk factor for microvascular complications. This underlines the urgent need of aggressive screening for early detection of microvascular and macrovascular complications and also to prevent or retard the progression of complications. Beyond screening, educating patients regarding diabetes related complications must be started to encourage earlier medical consultation.

## Figures and Tables

**Table 1:** Clinical and biochemical characteristics of patients with newly diagnosed diabetes mellitus (*n* = 449).

Variables	No microvascular complication (*n* = 368)	Any one of the three complications (*n* = 81)	*P* value
Age^*^	49.3 ± 10.2	56.0 ± 8.9	<0.0001^a^
Duration of diabetes (years)^#^	0.2 (0.1–0.4)	0.16 (0.1–0.3)	0.211^b^
Gender *n* (%) male	173 (47)	33 (41)	0.326^c^
Gender *n* (%) female	195 (53)	48 (59)	
BMI (Kg/m^2^)^*^	27.4 ± 5.2	27.2 ± 4.5	0.748^a^
Waist circumference (cm)^*^	96.5 ± 13.1	96.9 ± 12.9	0.803^a^
Hypertension *n* (%)	196 (53)	35 (43)	0.112^c^
TC (mg/dL)^*^	200 ± 53	193 ± 58	0.313^a^
LDL C (mg/dL)^*^	117 ± 37	110 ± 43	0.158^a^
HDL C (mg/dL)^*^	42 ± 10	42 ± 8	0.724^a^
TG (mg/dL)^#^	103 (37–165)	150 (92–198)	0.005^b^
HbA1c^*^	8.2 ± 2.3	9.1 ± 2.5	0.001^a^
Serum creatinine^#^	0.8 (0.7–0.9)	0.8 (0.7–0.9)	0.122^b^
Smoking *n* (%)	48 (13)	7 (9)	0.364^c^
Alcohol *n* (%)	69 (19)	10 (12)	0.226^c^
Socioeconomic status			
Upper *n* (%)	45 (12)	9 (11)	0.235^c^
Middle *n* (%)	250 (68)	49 (61)	
Lower *n* (%)	73 (20)	23 (28)	

^*^Expressed as mean ± standard deviation and ^#^median interquartile range. ^a^Analyzed using independent sample test, ^b^analyzed using Mann-Whitney test, and ^c^analyzed using chi-square test; TC: total cholesterol, LDL-C: low density lipoprotein cholesterol, HDL-C: high density lipoprotein cholesterol, TGs: triglycerides, and HbA1c: glycated hemoglobin.

**Table 2:** Risk factors for microvascular complications in patients with newly detected diabetes mellitus.

Risk factor	Any microvascular complication		Neuropathy		NPDR		PDR		Nephropathy	
	Odds ratio (95% CI)	*P* value	Odds ratio (95% CI)	*P* value	Odds ratio (95% CI)	*P* value	Odds ratio (95% CI)	*P* value	Odds ratio (95% CI)	*P* value
Age	1.06 (1.03–1.08)	<0.01^*^	1.08 (1.04–1.12)	<0.01^*^	1.02 (0.99–1.06)	0.172	1.00 (0.95–1.05)	0.851	1.00 (0.95–1.06)	0.816
Female gender	1.17 (0.71–1.93)	0.518	1.36 (0.67–2.77)	0.383	0.99 (0.45–2.21)	0.993	2.85 (0.91–8.89)	0.070	0.06 (0.01–0.51)	0.01^*^
BMI	0.99 (0.94–1.04)	0.746	0.98 (0.91–1.05)	0.621	0.97 (0.89–1.05)	0.490	1.05 (0.96–1.15)	0.279	0.99 (0.89–1.10)	0.910
SBP	1.01 (0.98–1.02)	0.623	0.98 (0.96–1.01)	0.379	1.01 (0.99–1.03)	0.224	1.01 (0.97–1.03)	0.834	1.01 (0.98–1.04)	0.470
HbA1c	1.24 (1.12–1.37)	0.047^*^	1.01 (0.84–1.2)	0.897	1.33 (1.16–1.53)	0.045^*^	1.88 (1.13–2.34)	0.034^*^	0.92 (0.70–1.22)	0.593
Total cholesterol	0.99 (0.98–1.01)	0.489	0.98 (0.97–0.99)	0.013	1.00 (0.99–1.01)	0.254	1.00 (0.99–1.01)	0.864	1.00 (0.99–1.01)	0.607
LDL	0.99 (0.98–1.01)	0.483	0.98 (0.97–0.99)	0.011	1.00 (0.99–1.01)	0.393	1.00 (0.99–1.01)	0.486	1.00 (0.99–1.02)	0.327
HDL	0.99 (0.96–1.02)	0.681	0.99 (0.94–1.04)	0.728	0.98 (0.93–1.02)	0.408	1.01 (0.95–1.07)	0.697	0.99 (0.92–1.06)	0.882
TG	1.01 (1.00–1.02)	0.011^*^	1.01 (1.0–1.02)	0.05^*^	1.01 (1.00–1.02)	0.02^*^	0.99 (0.98–1.01)	0.764	1.01 (1.00–1.02)	0.032^*^
Serum creatinine	0.91 (0.54–1.55)	0.753	0.78 (0.27–2.27)	0.659	0.26 (0.08–0.84)	0.024	0.73 (0.20–2.70)	0.643	1.55 (1.26–2.59)	0.047^*^
Smoking	0.73 (0.33–1.59)	0.434	0.89 (0.32–2.46)	0.825	0.89 (0.25–3.09)	0.854	0.42 (0.05–3.25)	0.408	1.27 (0.27–5.93)	0.761
Alcohol	0.56 (0.27–1.15)	0.118	1.02 (0.42–2.46)	0.962	0.17 (0.02–1.31)	0.091	0.67 (0.33–2.84)	0.853	0.82 (0.18–3.80)	0.805

^*^Statistically significant; the odds ratio for each variable or factors was adjusted for risk factors like age, HbA1c, PPG, HDL, BMI, LDL, TC, smoking, and alcohol status; TC: total cholesterol, LDL-C: low density lipoprotein cholesterol, HDL-C: high density lipoprotein cholesterol, TGs: triglycerides, HbA1c: glycated hemoglobin, NPDR: nonproliferative diabetic retinopathy, and PDR: proliferative diabetic retinopathy.

**Table 3:** Prescribing pattern of antihyperglycemic and concurrent medication in patients with newly diagnosed diabetes mellitus (*n* = 449).

Class of antidiabetic medication	Percentage prescribed *n* (%)
Biguanides	333 (74)
Sulfonylureas	175 (39)
Thiazolidinediones	29 (6)
Insulin	167 (37)
Antihypertensives	176 (39)
Antiplatelets	29 (6)
Lipid lowering	47 (10)
Combination	
Insulin alone	47 (11)
Insulin + OHA	120 (27)
OHA	359 (80)
OHA monotherapy	196 (44)
Biguanides	175 (39)
Sulfonylureas	21 (5)
OHA dual therapy	147 (33)
Biguanides + sulfonylureas	133 (30)
Biguanides + thiazolidinediones	8 (2)
Sulfonylureas + thiazolidinediones	5 (1)
OHA polytherapy	16 (4)
Sulfonylureas + biguanides + thiazolidinediones	16 (4)

OHA: oral hypoglycemic agents.
